# Subclinical Hypothyroidism as a Risk for Coronary Artery Bypass Grafting

**DOI:** 10.1155/cdr/9940175

**Published:** 2026-01-12

**Authors:** Ricardo Mendes Martins, Felipe José M. Pittella, Beatriz C. de Oliveira, Bernardo G. S. Lima, Caio C. Ferreira, Aline D. Pereira, Wolney A. Martins, Rubens A. C. Filho, Giovanna A. B. Lima

**Affiliations:** ^1^ Department of Internal Medicine, Federal Fluminense University, Niteroi, Rio de Janeiro, Brazil, uff.br; ^2^ Coronary Disease Unit, National Institute of Cardiology, Rio de Janeiro, Rio de Janeiro, Brazil, cardiologia.org.mx; ^3^ Institute of Geography, Rio de Janeiro State University (UERJ), Cabo Frio, Rio de Janeiro, Brazil, uerj.br

## Abstract

**Background:**

The relationship between hypothyroidism and cardiovascular disease is well established. However, data on subclinical hypothyroidism (SH) and its impact on major adverse cardiovascular events (MACEs) and postoperative complications following coronary artery bypass grafting (CABG) remain limited. This study was aimed at evaluating whether SH is associated with an increased risk of these outcomes.

**Methods:**

From 2010 to 2019, 863 patients who underwent CABG for cardiovascular diseases at a reference center were included. The primary outcomes included MACE (composite and individual events: all‐cause death, cardiovascular death, stroke, acute myocardial infarction [AMI], and new revascularization) and postoperative complications, including atrial fibrillation (AF), pleural effusion (PLE), pericardial effusion (PCE), infections at any site (IASs), and mediastinitis.

**Results:**

SH patients had higher rates of MACE (20.3% vs. 8.2%, *p* = 0.001), MACE 4p (22.0% vs. 12.9%, *p* = 0.002), and stroke (10.2% vs. 3.0%, *p* = 0.013) than those of euthyroid patients. No significant differences were observed in all‐cause death, cardiovascular death, AMI, or new revascularization. Postoperative complications were also more frequent in the SH group: AF (18.6% vs. 9.7%, *p* = 0.043), PLE (52.6% vs. 19.2%, *p* < 0.0001), PCE (20.3% vs. 7.8%, *p* = 0.03), and IAS (28.8% vs. 16.9%, *p* = 0.032). However, no significant difference was noted in the incidence of mediastinitis.

**Conclusions:**

SH patients who underwent CABG had a higher frequency of MACE and postoperative complications than those of euthyroid patients.

## 1. Introduction

Subclinical hypothyroidism (SH) is defined as an elevated serum thyroid‐stimulating hormone (TSH) level with normal serum free thyroxine (fT4) levels [1]. SH patients may experience nonspecific symptoms or remain completely asymptomatic, making diagnosis reliant on laboratory testing [2]. This condition is most frequently observed in older Caucasian women, with a prevalence ranging from 4% to 15% [3, 4]. Relationships between SH and coronary heart disease, cardiovascular death [5], heart failure [6], and stroke [7] have been described previously. However, literature regarding the association between SH and postoperative complications of coronary artery bypass grafting surgery (CABG) is scarce. Previous studies on SH and postoperative outcomes in patients undergoing CABG have shown conflicting results. Park et al. [8] reported an increased risk of atrial fibrillation (AF) in SH patients, whereas Zhao et al. [9] found no significant differences, and Komatsu et al. [10] suggested a potential protective effect of SH in cardiac surgery. Regarding major cardiovascular events, some studies have reported higher rates of revascularization and mortality in SH patients [11–13]. This study was aimed at evaluating the association between SH and the frequency of postoperative complications and major cardiovascular events in patients undergoing CABG.

## 2. Patients and Methods

**Figure 1:**
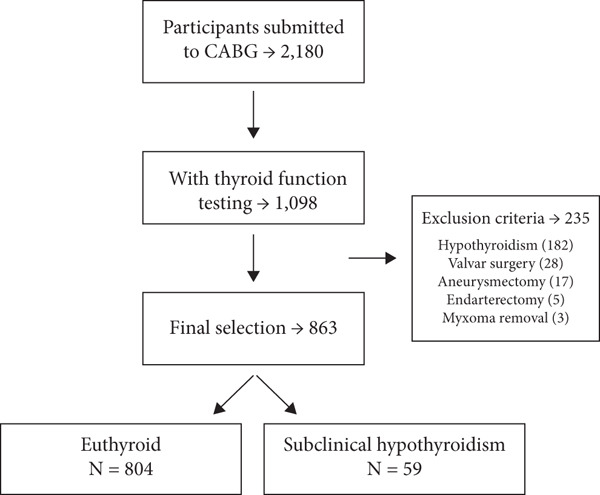
Patient selection flowchart.

This retrospective cohort study was conducted at a reference center for treating cardiovascular diseases. Over a 10‐year period [2010–2019], 2180 patients who underwent CABG were recruited. The inclusion criteria included patients aged ≥ 18 years with at least one TSH measurement, regardless of sex. Patients with a history of thyroid disease, prior use of thyroid hormone preparations, or those who underwent additional cardiac procedures in addition to isolated CABG (such as valve replacement) were excluded. A total of 1098 patients were initially recruited, and after applying the exclusion criteria, 863 patients were included in the analysis. The samples were divided into two groups: SH (*n* = 59) and euthyroid (*n* = 804) (Figure 1). Classification into the SH and euthyroid groups was performed according to TSH reference ranges defined by a large national study, which established age‐specific thresholds for TSH levels [14].

Sociodemographic and anthropometric data (age, body mass index [BMI], sex, and skin color) were collected in addition to classical risk factors associated with coronary artery disease: diabetes, hypertension, dyslipidemia, and smoking. Major cardiovascular events encompassed all‐cause mortality, cardiovascular death, nonfatal acute myocardial infarction (AMI), nonfatal stroke, and need for new revascularization. Postoperative complications included AF, pleural effusion (PLE), pericardial effusion (PCE), infections of any site (IASs), and mediastinitis. Hypertension was defined by blood pressure readings ≥ 140/90 mmHg or current use of antihypertensive medication. Diabetes was diagnosed by fasting plasma glucose levels ≥ 126 mg/dL and glycated hemoglobin (HbA1c) levels ≥ 6.5*%*, as well as by patient‐reported history of diabetes or use of antidiabetic medications. Dyslipidemia was identified by elevated levels of total cholesterol (≥ 200 mg/dL), low‐density lipoprotein (≥ 160 mg/dL), triglycerides (≥ 150 mg/dL), decreased high‐density lipoprotein (< 40 mg/dL for men and < 50 mg/dL for women), or the previous use of statins. BMI was calculated as weight in kilograms divided by height in meters squared (kg/m^2^). AF was defined by the presence of irregular heart rhythms without P waves on an electrocardiogram (ECG) or documented history of AF requiring pharmacological or electrical intervention. PLE was diagnosed through imaging (chest X‐ray, ultrasound, or computed tomography [CT]) indicating fluid accumulation within the pleural space, while PCE was identified by echocardiographic evidence of fluid in the pericardial cavity. IASs were considered when there was clinical, microbiological, or radiological evidence of infection, including but not limited to respiratory, urinary, or surgical wound infections. Mediastinitis was diagnosed based on clinical signs and symptoms, radiologic evidence, and microbiological culture. Nonfatal AMI was diagnosed by typical chest pain, elevated cardiac biomarkers (e.g., troponins), and/or ECG changes indicative of ischemia. Nonfatal stroke was identified by the sudden onset of compatible neurological symptoms (such as unilateral weakness, speech disturbances, or visual field deficits), confirmed by imaging (CT or magnetic resonance image) showing cerebral infarction or hemorrhage. Cardiovascular death was defined as fatal AMI, fatal stroke, or sudden cardiac death. New revascularization was deemed necessary if percutaneous coronary intervention (PCI) or CABG was performed due to symptomatic or angiographically significant coronary artery disease during the follow‐up period. The CABG procedure was performed by the local team of cardiac surgeons. Additionally, the sum of the following outcomes was also assessed: nonfatal AMI, nonfatal stroke, and death due to AMI or stroke (major adverse cardiovascular events (MACEs)). Lastly, the sum of MACE with the need for new revascularization was also evaluated (Four points MACE [MACE 4p]). Serum TSH and fT4 concentrations were measured by chemiluminescent immunoassay using commercial kits (Architect TSH, Abbott, Abbot Park, Illinois).

The study was approved by the Local Research Ethics Committee (CAAE, Registered Project Number 43887621930015272).

## 3. Statistical Analysis

Normality of numerical data was assessed using the Kolmogorov–Smirnov test. Variables with a normal distribution were expressed as mean ± standard deviation, while nonnormally distributed variables were presented as median and interquartile range (IQR). Comparisons between patients with and without SH were conducted using the Student′s *t*‐test or Mann–Whitney test, as appropriate. Categorical variables were expressed as absolute frequencies and percentages, and associations with SH were analyzed using the chi‐square test or Fisher′s exact test. Relative risk (RR) was calculated to evaluate risk factors associated with SH; an RR > 1 indicates a risk factor, RR < 1 a protective factor, and RR = 1 no association. Binary logistic regression was used to assess associations between patient characteristics and SH. All analyses were performed using IBM SPSS Statistics, Version 20 (IBM Corp., Armonk, NY, United States), with a significance level set at 0.05. Effect sizes (*d*) and confidence intervals were used to quantify the magnitude of the significant outcomes.

## 4. Results

The prevalence of SH in this cohort was 6.84%. Both groups were comparable in terms of basal characteristics as well as the presence of risk factors classically associated with cardiovascular diseases such as diabetes, hypertension, dyslipidemia, and smoking frequency (Table 1). A significant difference was found in TSH levels, which was higher in SH groups when compared with euthyroid (*p* < 0.0001; 6.0 (1.73) vs. 1.82 (1.4); *d* = 3.68; 95% CI: 3.36–4.00—large effect). Considering intraoperative parameters, there was no difference in cardiopulmonary bypass time (90 ± 34 vs. 85 ± 27 min, *p* = 0.521) and aortic clamping time (76 ± 32 vs. 78 ± 38 min, *p* = 0.834) between the euthyroid and the SH groups, respectively (Table 1). Similarly, the proportion of patients undergoing CABG without cardiopulmonary bypass and aortic clamping was not significantly different between the groups (10.2% vs. 9.2%; odds ratio: 1.12; 95% CI: 0.45–2.79; *p* = 0.815). The participants′ average follow‐up duration was 51.7 ± 35.9 months and the maximum follow‐up duration was 143.7 months. The frequency of MACE was higher in the SH group when compared to the euthyroid group (20.3% vs. 8.2%, *p* = 0.001; *d* = 0.40; 95% CI: 0.14–0.67—small effect) as well the frequency of MACE 4p (22.0% vs. 12.9%, *p* = 0.002; *d* = 0.24; 95% CI: 0.00–0.50—small effect). Stroke occurred in 3.5%, with a higher frequency in the SH group when compared to the euthyroid group (10.2% vs. 3.0%, *p* = 0.013; *d* = 0.39; 95% CI: 0.13–0.66—small effect). The frequency of AMI did not differ significantly between the SH and euthyroid groups (5.1% vs. 2.4%, *p* = 0.186). Cardiovascular mortality rates were also similar between the SH and euthyroid groups (5.1% vs. 2.9%, *p* = 0.414). Additionally, no significant difference was observed in the frequency of new revascularization procedures between the SH and euthyroid groups (1.7% vs. 4.7%, *p* = 0.511) (Table 2).

**Table 1:** Baseline characteristics of participants.

	**Euthyroid no. (%)**	**Subclinical hypothyroidism no. (%)**	**p** **value**
Patients	804	59	
Age (years), median (IQR)	62.5 (9.0)	62.0 (8.8)	0.645
BMI (kg/m^2^), median (IQR)	27.3 (4.5)	27.6 (4.1)	0.868
Men, *n* (%)	577 (71.8)	36 (61.0)	0.101
Skin color			
White	480 (59.7)	33 (55.9)	0.932
Brown	213 (26.5)	18 (30.5)	
Black	82 (10.2)	6 (10.2)	
Not informed	29 (3.6)	2 (3.4)	
Diabetes mellitus, *n* (%)	419 (52.1)	35 (59.3)	0.345
Hypertension, *n* (%)	781 (97.1)	59 (100.0)	0.598
Dyslipidemia, *n* (%)	781 (97.1)	59 (100.0)	0.715
Smoking, *n* (%)	422 (52.5)	31 (52.5)	1.000
Need for cardiopulmonary bypass, *n* (%)	730 (90.8)	53 (89,8)	0.815
Cardiopulmonary bypass (min)	90 (34)	85 (27)	0.521
Aortic clamping (min)	76 (32)	78 (38)	0.834
TSH (mU/L), median (IQR)	1.82 (1.4)	6.0 (1.73)	**< 0.0001**
fT4 (ng/dL), median (IQR)	1.10 (0.3)	1.07 (0.30)	0.383

*Note:* Values are expressed as median (range interquartile) or number (%), except for age, BMI, cardiopulmonary bypass, aortic clamping, TSH, and fT4, which are expressed as medians plus interquartile ranges. Mann–Whitney test. The bold formatting was used to emphasize that the only difference between the groups was the TSH level.

Abbreviations: BMI, body mass index; fT4, free thyroxin; TSH, thyroid stimulant hormone.

**Table 2:** Major cardiovascular events in subclinical hypothyroidism patients compared to euthyroid patients.

	**Total,** **n** = 863 **(%)**	**Euthyroid,** **n** = 804 **(%)**	**Subclinical hypothyroidism,** **n** = 59 **(%)**	**p** **value**
Acute myocardial infarct, *n* (%)	22 (2.5)	19 (2.4)	3 (5.1)	0.186
Stroke, *n* (%)	30 (3.5)	24 (3.0)	6 (10.2)	**0.013**
Cardiovascular death, *n* (%)	26 (3.0)	23 (2.9)	3 (5.1)	0.414
New revascularization, *n* (%)	39 (4.5)	38 (4.7)	1 (1.7)	0.511
MACE, *n* (%)	78 (9.0)	66 (8.2)	12 (20.3)	**0.001**
MACE 4p, *n* (%)	117 (13.5)	104 (12.9)	13 (22.0)	**0.002**
All cause deaths, *n* (%)	58 (6.7)	53 (6.6)	5 (8.5)	0.786

*Note:* Fisher′s exact test (except for the MACE chi‐square test). Values shown in bold refer to outcomes that showed differences between the groups.

Abbreviation: MACE, major adverse cardiovascular event.

AF was more frequent in the SH than in euthyroid group (18.6% vs. 9.7%, *p* = 0.043; *d* = 0.30; 95% CI: 0.03–0.57—small effect). The frequency of PCE and PLE was higher in the SH compared with euthyroid group (20.3% vs. 7.8%, *p* = 0.003; *d* = 0.45; 95% CI: 0.18–0.71—small effect and 52.5% vs. 19.2%, *p* < 0.0001; *d* = 0.83; 95% CI: 0.56–1.10—large effect 1, respectively). The incidence of IAS was also increased in the SH group (28.8% vs. 16.9%, respectively, *p* = 0.032; *d* = 0.31; 95% CI: 0.05–0.58—small effect). The skin (wound infection) was among the most frequently affected sites (41.8% of all infections), followed by the respiratory (32.7%) and the urinary tract (19.0%). Mediastinitis (11.9% vs. 6.5%, *p* = 0.112) did not show significant difference between groups (Table 3). In the multivariate analysis, adjusting for age, sex, BMI, and relevant comorbidities, SH remained an independent predictor of stroke (OR 3.68; *p* = 0.006) and AF (OR 2.15; *p* = 0.031; Table 4).

**Table 3:** Postoperative complications in subclinical hypothyroidism patients compared to euthyroid patients.

	**Total** **n** = 863 **(%)**	**Euthyroid** **n** = 804 **(%)**	**Subclinical hypothyroidism** **n** = 59 **(%)**	**p** **value**
Atrial fibrillation, *n* (%)	89 (10.4)	78 (9.7)	11 (18.6)	**0.043**
Serous effusions, *n* (%)	230 (26.7)	193 (24.0)	37 (62.7)	**< 0.0001**
Pleural effusion, *n* (%)	185 (21.4)	154 (19.2)	31 (52.5)	**< 0.0001**
Pericardial effusion, *n* (%)	75 (8.7)	63 (7.8)	12 (20.3)	**0.003**
Infection of any site, *n* (%)	153 (17.7)	136 (16.9)	17 (28.8)	**0.032**
Mediastinitis, *n* (%)	59 (6.8)	52 (6.5)	7 (11.9)	0.112

*Note:* Values are expressed as number (%); Fisher′s exact test. Values shown in bold refer to outcomes that showed differences between the groups.

**Table 4:** Multivariate logistic analysis to evaluate associations between patient characteristics, atrial fibrillation, and stroke.

**Patient characteristics**	**Atrial fibrillation**		**Stroke**	
	**OR (95% CI)**	**p** **value**	**OR (95% CI)**	**p** **value**
Age^a^	1.042 (1.015–1.071)	0.003 ^∗^	0.991 (0.956–1.033)	0.651
Sex^b^	0.727 (0.426–1.195)	0.224	2.083 (0.855–6.228)	0.139
Skin color^c^	1.130 (0.619–2.066)	0.989	0.380 (0.266–0.543)	0.099
Diabetes mellitus^c^	0.740 (0.471–1.153)	0.187	1.471 (0.708–3.126)	0.303
Hypertension^c^	1.114 (0.995–1.247)	0.062	1.036 (0.968–1.108)	0.305
Dyslipidemia^c^	0.541 (0.198–1.899)	0.274	1.035 (0.960–1.116)	0.986
Smoking^c^	1.057 (0.681–1.647)	0.805	2.166 (1.010–5.032)	0.056
BMI	1.016 (0.964–1.070)	0.542	1.024 (0.935–1.114)	0.595
Need for cardiopulmonary bypass^c^	1.217 (0.578–2.987)	0.634	0,625 (0.235–2.164)	0.394
Subclinical hypothyroidism^c^	2.154 (1.025–4.184)	0.031 ^∗^	3.679 (1.318–8.863)	0.006 ^∗^
Atrial fibrillation^c^	—	—	2.332 (0.842–5.553)	0.073
Stroke^c^	2.332 (0.842–5.553)	0.073	—	—

*Note:* Reference variable were those that presented the highest frequency.

^a^Male sex as a reference variable.

^b^White color as a reference variable.

^**c**^Yes as a reference variable.

^∗^Significant association (*p* < 0.05).

We also evaluated the RR of the complications in this cohort. The RR of AF (1.99), PLE (4.06), PCE (2.68), IAS (1.88), and stroke (3.14) were significantly higher in the SH group (Table 5).

**Table 5:** Relative risks of postoperative complications in subclinical hypothyroidism patients compared to euthyroid patients.

	**Relative risk**	**IC 95%**	**p** **value**
Atrial fibrillation	1.99	1.07–3.70	**0.043**
Pleural effusion	4.06	2.50–6.59	**< 0.0001**
Pericardial effusion	2.68	1.49–4.83	**0.003**
Infection of any site	1.88	1.10–3.21	**0.032**
Stroke	3.14	1.47–6.73	**0.013**

*Note:* Fisher’s exact test. Values shown in bold refer to outcomes that showed differences between the groups.

Abbreviation: IC 95%, confidence interval 95%.

## 5. Discussion

In the present study, the two groups were similar in terms of baseline characteristics, cardiovascular risk factors, and intraoperative parameters. SH patients who underwent CABG had a higher risk of major cardiovascular events (stroke, MACE, and MACE 4p) and other postoperative complications (AF, PCE, PLE, and IAS) than those with normal thyroid function.

The relationship between SH and cardiovascular diseases, such as coronary heart disease, cardiovascular death [5], heart failure [6], stroke [7], and AF [15], has been well documented. However, there is limited literature on the association between SH and postoperative complications following CABG.

A significant difference was observed in composite outcomes, such as MACE and MACE 4p, primarily driven by the higher frequency of stroke in the SH group, which had a RR of 3.14. In the multivariate analysis, SH remained an independent predictor of stroke. These results suggest that the association between SH and stroke is not fully explained by traditional cardiovascular risk factors, supporting a potential direct pathophysiological role of SH in cerebrovascular events. Considering other major cardiovascular events, the absolute numbers of AMI, cardiovascular deaths, and all‐cause deaths were higher in the SH group; however, the differences were not statistically significant.

These findings are consistent with those of the three previous studies. A retrospective South Korean study (2018) involving 222 euthyroid individuals and 36 SH patients reported an increased need for new revascularization over a follow‐up period of 8.2 years. However, it found no differences in “hard outcomes” such as stroke, AMI, or mortality [11]. The present study comprised a larger sample size of 863 patients. A second study, published in 2020 by the same group and institution, retrospectively evaluated 461 participants, including 395 euthyroid individuals and 66 SH patients, with an average follow‐up of 7.6 years. Significant differences were observed in important outcomes, such as cardiovascular and all‐cause mortality, but there was no difference in the occurrence of stroke or MACE [12]. Finally, a large Chinese cohort study (2023) including 1088 patients, equally divided between the euthyroid and SH groups, observed an increase in cardiovascular mortality, MACE, and angina pectoris [13].

Postoperative AF after cardiac surgery is a complication that occurs in 15%–40% of cases [15–17]. Its occurrence results in a 15%–25% reduction in cardiac output [18], tachycardia, hypotension, heart failure, increased risk of cardioembolism [19], and longer hospitalization time, consequently increasing hospital costs [20, 21]. The most associated risk factors for postoperative AF in cardiac surgery include age, male sex, history of AF, valvular surgeries (especially mitral stenosis), pulmonary diseases, and right coronary artery obstruction [18, 22, 23]. The relationship between thyroid dysfunction and the postoperative period after cardiac surgery remains poorly understood. Lowered triiodothyronine (T3) levels in patients undergoing CABG [24] are associated with an increased risk of postoperative AF [25, 26]. This reduction is an adaptive mechanism to the surgical procedure in all patients; however, in SH patients, this phenomenon may be more pronounced and potentially more deleterious. T3 replacement could help mitigate these complications [27]. A South Korean study (2009) prospectively evaluated 222 euthyroid patients undergoing CABG and compared them with 36 SH patients, finding a positive association between SH and AF occurrence [8]. In contrast, a Chinese study (2021) that evaluated 1090 participants concluded that the rate of AF after CABG surgery was quite similar between the groups, at approximately 20% [9]. In 2022, Joe et al. evaluated 800 patients who were subdivided into three groups (euthyroid, SH, and isolated low T3) and found a higher prevalence of AF in the latter two groups [28]. Interestingly, a 2018 study demonstrated that overt hypothyroidism is a protective factor against postoperative AF after cardiac surgery. Nonetheless, it is important to note that over 60% of cases involved valvular surgery [10]. Recently, a meta‐analysis of 3445 patients, 851 with SH, demonstrated an increased risk of perioperative and long‐term general mortality, length of hospital stay, and renal complications in SH patients. No differences were observed in relation to the frequency of AF [29].

Our study included a large number of participants with an overall occurrence of AF in approximately 10.3% of patients, which was below the historical average [17]. This may be partly explained by the exclusion of patients who underwent concomitant valve procedures during CABG, as well as those with a history of chronic AF [28]. A statistically significant difference was observed between the groups, with an incidence of 18.6% in the SH group compared to 9.7% in patients without thyroid dysfunction, corresponding to a RR of 1.99. Although this finding suggests an association between SH and the outcome, its clinical significance should be interpreted with caution given the wide confidence intervals, which reflect uncertainty about the exact magnitude of the effect. In the multivariate analysis, SH remained an independent predictor of AF. These results indicate that the observed associations cannot be fully explained by traditional risk factors, supporting the hypothesis of a direct pathophysiological role of SH in arrhythmogenesis.

The relationship between hypothyroidism and serous effusion, especially PCE, was first described almost a century ago [30]. It is related to the degree of TSH elevation, has a slow onset, is rich in proteins and glycosaminoglycans, and rarely causes cardiac tamponade [31]. There are limited data on the incidence of these complications in SH patients, with few reported cases in the literature [32–34]. In the context of postoperative CABG, PLE is a complication that occurs in up to 90% of cases. While it is typically small and does not require specific treatment, it can, in some cases, affect pulmonary mechanics and delay extubation [35]. Similarly, PCE can occur in up to 85% of patients several days after a procedure. As with PLE, it is usually small and does not require intervention [36]. Whether SH is an additional risk factor for these complications in this patient group remains unclear. We evaluated this potential relationship and found a higher frequency of serous effusion in the SH group than in the euthyroid group. A PCE rate of 20.3% was observed in SH patients versus 7.8% in those with normal TSH levels (*p* = 0.003). Regarding PLE, a frequency of 52.5% was found in SH patients versus 19.2% in those with normal hormone function (*p* < 0.0001). Both complications were statistically significant, with RR of 4.06 for PLE and 2.68 for PCE. The combined percentage of serous effusions (PCE and PLE) was 72.9% in SH patients, compared to 27% in euthyroid patients.

This study found an increase in the frequency of IAS in the SH group (28.8%) compared to the control group (16.9%) and a RR of 1.88. The most affected infectious sites were surgical wounds (41.8%), the respiratory tract (32.7%), the urinary tract (19.0%), and the bloodstream (4.6%). However, when considering mediastinitis alone, a complication commonly associated with the postoperative period of thoracic surgeries, there was no significant difference between the groups (11.9% in the SH group vs. 6.5% in the euthyroid group). Our findings are consistent with an arm of the SYNTAX trial (2020) involving 1800 participants, which reported that the most common postoperative infections were surgical wound infections, followed by pneumonia and bloodstream infections [37]. The study also found that IAS after CABG were associated with increased long‐term mortality. However, although this association was statistically significant, the wide confidence intervals, comparable to those observed for AF, indicate that the clinical implications of this finding should be interpreted with caution. The relationship between thyroid hormones and the immune system is complex. Both T3 and fT4 actively participate in both innate and adaptive responses through genetic and nongenetic mechanisms [38]. However, data regarding the clinical effects of this interaction are limited, especially in mild thyroid disease patients.

This study has limitations inherent to its retrospective design. Its single‐center nature may limit the external validity of the findings. Notably, 21.3% of patients were lost to follow‐up within the first year, introducing potential bias, particularly in the assessment of long‐term outcomes such as MACE and mortality. Finally, transient TSH elevations cannot be entirely excluded, as most patients had only a single measurement. However, because TSH was assessed in an outpatient setting, the likelihood of nonthyroidal illness syndrome or perioperative stress influencing the results is minimized.

## 6. Conclusions

The presence of SH in patients undergoing CABG was associated with a higher frequency of major cardiovascular events and postoperative complications.

## Conflicts of Interest

The authors declare no conflicts of interest..

## Funding

No funding was received for this manuscript.

## Data Availability

Research data are not shared.
